# The unmet need for COVID-19 treatment in immunocompromised patients

**DOI:** 10.1186/s12879-022-07918-x

**Published:** 2022-12-12

**Authors:** D’Abramo Alessandra, Vita Serena, Nicastri Emanuele

**Affiliations:** National Institute for Infectious Diseases “Lazzaro Spallanzani” IRCCS, Via Portuense 292, 00149 Rome, Italy

**Keywords:** Immunocompromised patients, Severe and/or prolonged COVID-19, Passive immunotherapy, Antiviral therapy, Combined therapy

## Abstract

**Background:**

Immunocompromised (IC) patients are at increased risk of severe and/or prolonged COVID-19.

**Main text:**

The recent study by Scaglione et al., addresses the issue of IC outpatients with SARS-CoV-2 infection. Authors describe the real-life use of SARS-CoV-2 antivirals and/or monoclonal antibodies and the clinical benefit in high-risk COVID-19 patients. The study supports the use of early combination therapy in a subgroup of extremely high risk patients, and considers the combined strategy as a gold standard regimen to both increase the effectiveness of early treatment, especially in IC individuals, and, reduce the emergence of SARS-CoV-2 escape mutants.

**Conclusion:**

A tailored and standardised therapeutic approach in case of IC out and inpatients with SARS-CoV-2 infection is needed.

## Background

Immunocompromised (IC) patients with autoimmune, onco-haematological and/or neurological diseases, with negative or low titre serology against SARS-CoV-2 after natural infection and/or vaccination are at increased risk of severe and/or prolonged COVID-19 [1].

## Main text

IC patients often treated with anti-CD20 antibodies such as rituximab, or with congenital or acquired hypogammaglobulinemia, result in B lymphocyte depletion that occur within 72 h after administration, with an estimated recovery time of 6–9 months after the end of therapy and a return to normal levels observed after 9–12 months [2]. Fingolimod is another drug used for multiple sclerosis resulting in peripheral lymphocyte sequestration [3]. The COVID-19 pandemic has been and continues to be serious problem among IC patients, particularly for those treated with B-lymphocyte depletion biological agents, with a COVID-19 case fatality rate unacceptably high as 40% [4]. Indeed, immunocompromised patients may have clinical and virological evidence of persistent SARS-CoV-2 infection lasting longer than 21 days and/or more than two episodes of acute respiratory syndrome [5]. In this context, it is important to identify these patients early and establish an effective therapy. The European and Italian Drug Agency both recommend the use of antivirals (remdesivir, molnupinavir or nirmatrelvir/ritonavir) or monoclonal antibodies (MoAbs) against SARS-CoV-2 S-glycoprotein (sotrovimab or tixagevimab with cilgavimab) for early treatment of COVID-19 patients at high risk of disease progression [6, 7]. To date, there are no precise recommendations for the treatment of IC patients with acute or prolonged SARS-CoV-2 infection due to the absence of these patients in registration clinical trials [8–10]. However, there are no tailored indications for the treatment of IC patients with acute or prolonged SARS-CoV-2 infection. Passive immunotherapy, such as hyperimmune convalescent plasma and/or MoAbs, represents a source of exogenous specific antibodies in IC patients with primary or secondary humoral disorders [11, 12]. Recently, a number of small-case studies have demonstrated the efficacy of combined antiviral and MoAb treatments from both clinical and virological points of view in IC inpatients with prolonged SARS-CoV-2 infection [13]. The recent study by Scaglione et al., addresses the issue of IC outpatients with SARS-CoV-2 infection. Authors describe the real-life use of SARS-CoV-2 antivirals and/or MoAbs and the clinical benefit in high-risk COVID-19 patients in term of the hospitalisation rate. Two hundred eighty-eight patients were analysed and treated according to tailored still unvalidated diagnostic and decision making algorithm; 94/288 (32.6%) patients were treated with MoAb monotherapy, 171/288 (59.4%) patients were treated with antivirals, and 23/288 (8%) patients received a combined therapy (antivirals and MoAbs). The study support the use of early combination therapy in a subgroup of extremely high risk patients, and considers this combined strategy as a gold standard regimen to both increase the effectiveness of early treatment, especially in IC individuals, and, reduce the emergence of SARS-CoV-2 escape mutants [14]. Combined antiviral and passive immunotherapy has already been used in a cohort of patients receiving anti-CD20 biological agents with prolonged SARS-CoV-2 infection, proving to be effective and safe in terms of reducing viral load, immunoactivation and case fatality rates [15], while there are no data on the combined use of antiviral and hyperimmune plasma obtained from convalescent COVID-19 patients. Although MoAbs have been largely used in patients with Delta variant SARS-CoV-2 infections, the current epidemiologic wave sustained by Omicron variants, raise concerns on residual antiviral efficacy and clinical benefit of sotrovimab and tixagevimab/cilgavimab [16].

## Conclusion

In the current covid-19 scenario, the risk of severe disease is higher in IC patients. The inclusion of such patients in registration clinical trials is crucial to support the few still sparse experience of combined therapy reported in literature. A tailored and standardised therapeutic approach in case of IC out and inpatients with SARS-CoV-2 infection at very high risk of clinical progression is needed.

## Data Availability

Not applicable.
